# Rapid Colloidal Gold Immunoassay for Pharmacokinetic Evaluation of Vancomycin in the Cerebrospinal Fluid and Plasma of Beagle Dogs

**DOI:** 10.3390/s23218978

**Published:** 2023-11-05

**Authors:** Yechang Guo, Shaofeng Wang, Peiyue Li, Pan Zhang, Wei Wang

**Affiliations:** 1 School of Integrated Circuits, Peking University, Beijing 100871, China; g.yech@pku.edu.cn (Y.G.); peiyueli@stu.pku.edu.cn (P.L.); panzhang1989@pku.edu.cn (P.Z.); 2 School of Engineering and Technology, China University of Geosciences (Beijing), Beijing 100083, China; 2102210006@email.cugb.edu.cn; 3 National Key Laboratory of Advanced Micro and Nano Manufacture Technology, Beijing 100871, China; 4 Beijing Advanced Innovation Center for Integrated Circuits, Beijing 100871, China

**Keywords:** colloidal gold immunoassay, pharmacokinetic study, test strip, vancomycin

## Abstract

Vancomycin (VAN), a glycopeptide antibiotic, is the preferred therapeutic agent for treating Gram-positive bacteria. Rapid and precise quantification of VAN levels in cerebrospinal fluid (CSF) and plasma is crucial for optimized drug administration, particularly among elderly patients. Herein, we introduce a novel clinical test strip utilizing colloidal gold competitive immunoassay technology for the expedient detection of VAN. This test strip enables the detection of VAN concentrations in clinical samples such as plasma within 10 min and has a limit of detection of 10.3 ng/mL, with an inhibitory concentration 50% (IC_50_) value of 44.5 ng/mL. Furthermore, we used the test strip for pharmacokinetic analysis of VAN in the CSF and plasma of beagle dogs. Our results provide valuable insights into the fluctuations of the drug concentration in the CSF and plasma over a 24 h period after a single intravenous dose of 12 mg/kg. The test strip results were compared with the results obtained via liquid chromatography–mass spectrometry methods, and the measured VAN concentrations in the CSF and plasma via both of the methods showed excellent agreement.

## 1. Introduction

Monitoring drug treatments for common infections among the elderly population is pivotal in healthcare practice [1]. Physiological changes, such as immunosenescence and the decline of liver and kidney functions, render older individuals more vulnerable to infections. Moreover, elderly patients often exhibit atypical symptoms, making the pathophysiological processes more complex than those of younger individuals. These complexities underscore the critical importance of meticulous medication management and monitoring. In elderly individuals, metabolic and excretory functions may be compromised, leading to prolonged drug retention times in the body. To maintain therapeutic drug levels in the body, physicians often require drug concentration monitoring to avoid underdosing or overdosing [2,3,4,5].

Vancomycin (VAN) is an essential therapeutic agent for combating Gram-positive bacterial infections, particularly methicillin-resistant *Staphylococcus aureus* (MRSA) [6,7]. Although MRSA is resistant to a wide array of antibiotics, including β-lactams and cephalosporins, it remains susceptible to VAN, which is the recommended first-line treatment for severe MRSA infections [8,9]. However, the clinical application of VAN poses challenges owing to its potential toxicity, which can manifest in severe adverse effects such as kidney injury [10]. Therefore, real-time assessment of VAN concentrations in serum is crucial for dosage adjustments to maximize therapeutic benefits and minimize adverse reactions [11,12,13].

Numerous methodologies have been developed and refined for VAN quantification in the field of analytical and clinical chemistry. These include high-performance liquid chromatography (HPLC) [14], liquid chromatography–mass spectrometry (LC–MS) [15,16,17,18], enzyme-linked immunosorbent assay (ELISA) [19], and electrochemical methods [20,21,22,23]. HPLC and LC–MS, while boasting unparalleled sensitivity and specificity, often come with a hefty price tag in terms of resource consumption. They necessitate sophisticated equipment, specialized operators, and extended processing times, making them less amenable to situations demanding rapid results. Similarly, while ELISA provides a robust platform for VAN detection, it also shares the limitations of extended processing times and resource intensiveness. Electrochemical methods, on the other hand, stand out for their capability to swiftly detect VAN, often meeting the pressing timelines of clinical demands. However, a significant hurdle with electrochemical methods is their sensitivity limitation, which is on the order of μg/mL [22,24,25]. 

Interestingly, the realm of immunodiagnostics offers a promising avenue. The competitive format of immunochromatographic strip assays, rooted in the specific binding between antibodies and antigens, presents several advantages over traditional methods, especially for the rapid screening of smaller analytes [26,27,28]. The virtues of these assays lie in their simplicity, user-friendliness, and cost-effectiveness [29,30,31,32]. They have proven their mettle in the real-time detection of myriad analytes, ranging from heavy metals [33], pharmaceutical drugs [34,35,36], and toxins [37,38,39], to hormones [40], serving a wide spectrum of applications. However, when it comes to VAN detection using immunological methods, the literature remains scant. Only a handful of studies have ventured into this space [41,42], and among these, the most impressive limit of detection (LOD) achieved to date stands at 69.2 ng/mL [42], signaling both the potential and challenges of this approach.

This study aims to develop and validate a colloidal gold immunoassay for rapid VAN quantification in clinical samples. Colloidal gold testing offers a balance of speed, cost-effectiveness, and miniaturization potential, making it an attractive option for point-of-care applications. Herein, we developed a VAN test strip based on this methodology and performed pharmacokinetic analyses in beagle dogs. The test results were further corroborated against data acquired through LC–MS, validating our assay as a reliable alternative for VAN monitoring.

## 2. Materials and Methods

### 2.1. Materials

Vancomycin hydrochloride (Cat. No. V105495), teicoplanin (Cat. No. T304131), dalbavancin (Cat. No. D329356), telavancin (Cat. No. T614373), oritavancin (Cat. No. O612552), amikacin (Cat. No. A169991.), and meropenem (Cat. No. P341828) were purchased from Aladdin Scientific Corp. (Shanghai, China). In addition to the antibiotics, other chemical reagents such as carbamazepine powder (Cat. No. C407797), potassium carbonate (K_2_CO_3_), hydrochloric acid (HCl), sodium chloride (NaCl), gold chloride (HAuCl_4_), trisodium citrate, bovine serum albumin (BSA), phosphate-buffered saline, Tris buffer, methanol, and formic acid were obtained from Aladdin Scientific Corp. (Shanghai, China). The VAN–BSA antigen and antibodies were sourced from Shenzhen KeJie Industrial Development Co., Ltd. (Shenzhen, China). Nitrocellulose (NC) membranes were purchased from Whatman (Dassel, Germany).

### 2.2. Solution Preparation

We prepared various solutions for this study. A 50% methanol aqueous solution was prepared by mixing 250 mL of methanol with an equivalent volume of ultrapure water. The mixture was then sonicated for 10 min at an ultrasonic power of 120 W using an ultrasonic machine (KM-23C) purchased from Guangzhou KeMing Clean Technology Co., Ltd. (Guangzhou, China). The solution was subsequently stored at 2–8 °C. Stock solutions of VAN hydrochloride and carbamazepine were prepared at concentrations of 1 mg/mL using 3 mg of VAN dissolved in 3000 μL of water and 2 mg of carbamazepine dissolved in 2000 μL of methanol, respectively. Both of the stock solutions were stored at 2–8 °C. Working solutions of VAN were prepared by diluting the stock solution to the concentrations of 50, 150, 300, 1000, 2000, 6000, 9000, and 10,000 ng/mL. Quality control solutions of VAN were prepared at concentrations of 150, 500, and 750 ng/mL. An internal standard solution of carbamazepine was prepared at a working concentration of 1 μg/mL. All solutions were stored at 2–8 °C. Finally, a 0.1% formic acid solution was prepared by adding 500 μL of formic acid to 500 mL of ultrapure water. The mixture was then sonicated for 10 min using an ultrasonic machine (KM-23C) from Guangzhou KeMing Clean Technology Co., Ltd. (Guangzhou, China) and stored at room temperature.

### 2.3. Preparation of Beagle Dog CSF and Plasma Samples

Animal Dosing and Sample Collection: The choice of using beagle dogs for the pharmacokinetic studies of VAN in CSF and plasma is driven by their physiological similarities to humans, which make them a suitable model for predicting human pharmacokinetics [43,44,45]. Three male beagle dogs, each weighing approximately 11 kg and aged 10 months, were utilized for pharmacokinetic studies. Both the acquisition of the beagle dogs and the sample collection were conducted by Beijing Sinogenetic Biotechnology Co., Ltd. (Beijing, China). The animal protocols employed for this research were reviewed and approved by Beijing Sinogenetic Biotechnology Co., Ltd. (XNY-20230524001). The beagle dogs were anesthetized and subsequently administered a single intravenous dose of a pre-prepared 50 mg/mL VAN hydrochloride solution at a dosage level of 12 mg/kg, with the injection time lasting approximately 2–3 s. CSF and plasma samples were collected at predetermined time points: predose (0 min) and postdose at intervals of 5 min, 10 min, 30 min, 1 h, 2 h, 4 h, 8 h, and 24 h. Before each sampling, propofol was administered to anesthetize the beagle dogs to a sedated state. CSF was collected from the cerebellar fossa of the beagles using a 1 mL syringe for puncture. Approximately 100 μL of CSF was collected in anticoagulant-free 1.5 mL centrifuge tubes at each time point. Similarly, ~0.5 mL of blood was collected in EDTA-K anticoagulant tubes from the pedal vein at each specified sampling interval.

CSF sample processing: The CSF samples were collected in centrifuge tubes and stored at 4 °C within 30 min of collection. These samples were then centrifuged at 3000 g for 10 min, after which the supernatant was collected and preserved at −80 °C for subsequent analysis.

Plasma sample processing: Similarly to CSF sample processing, the blood samples collected in anticoagulant tubes were stored at 4 °C within 30 min of collection. These samples were then centrifuged at 3000× *g* for 10 min, and the supernatant plasma was isolated and stored at −80 °C for future analysis.

### 2.4. Measurement of VAN Concentration Using the LC–MS Method

An HPLC–MS method was established to precisely quantify VAN levels in CSF and plasma samples collected from beagle dogs. The LC-MS instrument used was a TSQ Quantis Plus, purchased from Thermo Fisher Scientific Inc. (Waltham, MA, USA). The mass spectrometer featured an H-ESI ion source, operating in positive ion mode and utilizing a selected reaction monitoring detection paradigm. Instrumental parameters were meticulously set as follows: an ion spray voltage of 3500 V, an ion transfer–tube temperature of 325 °C, and a collision-induced dissociation gas pressure of 1.5 mTorr. Chromatographic separation was conducted using a Waters ACQUITY UPLC BEH C18 column (2.1 mm × 50 mm; 1.7 μm, Shanghai, China), operating at a flow rate of 0.4 mL/min. A mobile phase comprising 0.1% formic acid in water and pure acetonitrile was used. The autosampler and column temperatures were stringently regulated, and a specific elution gradient was applied to optimize separation. For sample preparation, 50 μL of either plasma or CSF was combined with a Karmasipin internal standard and acetonitrile. Subsequently, the mixture was vortexed and centrifuged to render it suitable for LC–MS analysis. The methodological rigor ensured a high degree of specificity and sensitivity in VAN quantification, effectively fulfilling the objectives of this study.

### 2.5. Preparation of Colloidal Gold and Labeled Antibodies

Colloidal gold nanoparticles, integral for inducing a visible color change at the test line of the assay strips, were synthesized by boiling 100 mL of a 0.01% HAuCl_4_ aqueous solution, followed by the addition of 1% sodium citrate aqueous solution [46,47,48]. The resulting nanoparticles exhibited a uniform diameter of ~30 nm. Subsequently, VAN antibodies were conjugated to these gold nanoparticles. The VAN antibodies used were monoclonal antibodies derived from mouse ascites with a molecular weight of 150 kD. They were purchased from Shenzhen KeJie Industrial Development Co., Ltd. (Shenzhen, China). The efficacy of this conjugation relied on a trifecta of forces: ionic attractions between the negatively charged gold particles and the positively charged proteins, hydrophobic interactions, and sulfur bonds, particularly from thiol groups that linked two regions of the Fc segment of the antibody to the gold surface. 

### 2.6. Preparation of Test Strips

A sequence of critical steps must be followed for fabricating colloidal gold immunochromatographic test strips. Initially, a substrate, typically comprising paper or a film, was precoated with antibodies specifically tailored for detecting the target molecule. Subsequently, colloidal gold nanoparticles were synthesized by reducing gold salts and coupled with antibodies. This coupling was facilitated by the combination of ionic, hydrophobic, and sulfur bond interactions. The resultant antibody-labeled colloidal gold solution was then applied to the designated test line area on the substrate. Then, a control line was established to validate the operational integrity of the strip. Following these applications, the strip underwent a drying process, was trimmed to manageable dimensions, and was hermetically sealed in protective packaging to mitigate the risk of environmental contamination.

### 2.7. Optical Measurement of Images

After adding the sample, the test strip either displayed two red lines (both the control and test lines) or just one red line (control line) within 10 min. The intensity of the color on the test line indicated the signal value. We quantified this signal using a smartphone camera in combination with the ImageJ software (Fiji ImageJ for Mac, 2.3.0 version) [49]. The images captured by the smartphone had an 8-bit depth. We used ImageJ to extract the gray values from lines drawn perpendicular to both the control and test lines. It is important to note that the gray values for the control and test lines are significantly lower than those of their surrounding areas, creating distinct valleys. Therefore, the effective signal, represented as *B*, is determined by the absolute difference in gray value between the test line and its adjacent area.

## 3. Results and Discussion

### 3.1. Mechanism of Analyte Detection via the Colloidal Gold Test Strips

**Figure 1:**
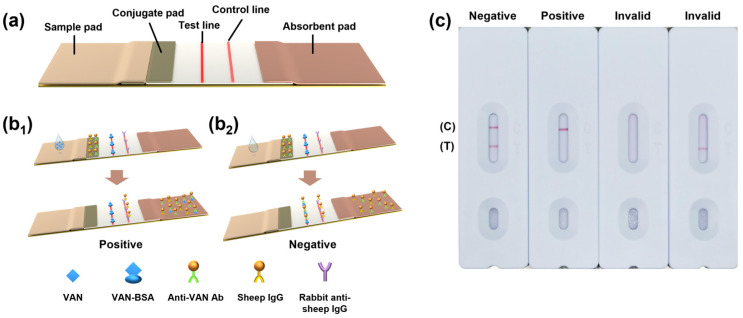
(**a**) Schematic of the colloidal gold test strip structure. (**b_1_, b_2_**) Working principle of the colloidal gold test strip. (**c**) Test strip results for negative, positive, and two types of invalid scenarios.

The architecture of the immunochromatographic strip is illustrated in Figure 1a and comprises four principal components: the sample pad, colloidal gold pad, NC membrane, and absorbent paper. The sample pad is made of glass fiber and serves multiple functions: it reduces the sample flow rate, ensures an even distribution of the sample onto the colloidal gold pad, filters out impurities, and adjusts the pH or viscosity of the sample. The colloidal gold pad, which also utilizes similar materials, functions as a repository to adsorb a set amount of gold-labeled conjugates. It continuously transfers the sample to the NC membrane and ensures the complete release of gold-labeled conjugates. The NC membrane, sourced from Whatman, immobilizes antibodies at the test and control lines, facilitating the immunological reaction with the sample and providing a visual readout of the results. The absorbent paper regulates the flow of the sample and employs capillary action to enable the movement of reagents across the membrane, not just onto it.

For small molecules like VAN, a competitive immunoassay is essential, as shown in Figure 1(b_1_,b_2_). The test line is coated with a competitive VAN–BSA antigen, and the control line is coated with an immunogold antibody. Upon application of the sample to the sample pad, the sample flows through the colloidal gold pad to interact with the gold-labeled antibodies. It then progresses through the test and control lines before being absorbed into the absorbent paper. The appearance of a visible line in the test area signifies a negative result for VAN (Figure 1(b1)). Conversely, the absence of a visible line suggests that a sufficient concentration of VAN is present, thereby occupying the antibody–gold nanoparticle binding sites and preventing them from binding to the VAN–BSA antigen, resulting in no visible line (Figure 1(b2)). Regardless of the presence or absence of VAN, a red control line should always appear when the test is operating correctly. Otherwise, the results are invalid. Figure 1c depicts the four scenarios that may arise during testing.

### 3.2. Characterization of Gold Nanoparticles

The synthesis of high-quality colloidal gold solutions is imperative for ensuring the diagnostic specificity and sensitivity of the immunochromatographic test strips. Colloidal gold, colloquially known as gold sol, comprises a suspension of gold nanoparticles generated via the chemical reduction of gold salts into their elemental form. Each gold nanoparticle comprises a central core of elemental gold, enveloped by stratified ionic layers. An inner layer of anionic species, specifically AuCl^2−^, is present adjacent to the core surface, whereas an outer layer of protons (H^+^) permeates the colloidal solution, stabilizing the nanoparticles in a homogeneously suspended state.

**Figure 2:**
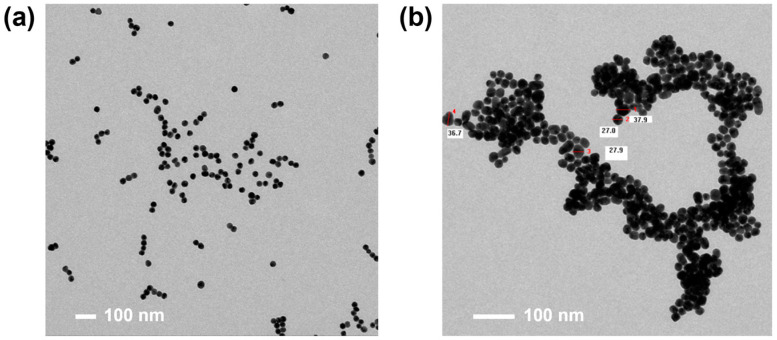
Transmission electron microscopy images of gold nanoparticles. (**a**) High-quality gold nanoparticles with good monodispersity, having an average diameter of 31.2 nm and a standard deviation of ±3.3 nm. (**b**) Lower-quality gold nanoparticles with poor monodispersity, showing an average diameter of 28.7 nm and a standard deviation of ±8.1 nm.

In the context of immunoassays, colloidal gold is frequently used to form complexes with immunologically reactive substances such as antigens or antibodies, collectively termed as immunogold complexes. The dimensional attributes and morphological characteristics of these complexes bear critical importance for the optimal performance of the assay. Figure 2a,b show the transmission electron microscopy images of high-quality and low-quality synthesized gold nanoparticles, respectively. The high-quality gold nanoparticles exhibit good monodispersity, centering around an average diameter of 31.2 nm with a standard deviation of ±3.3 nm. In contrast, the lower-quality gold nanoparticles show poor monodispersity with an average diameter of 28.7 nm and a standard deviation of ± 8.1 nm. 

### 3.3. Evaluation of Colloidal Gold Test Strips

**Figure 3:**
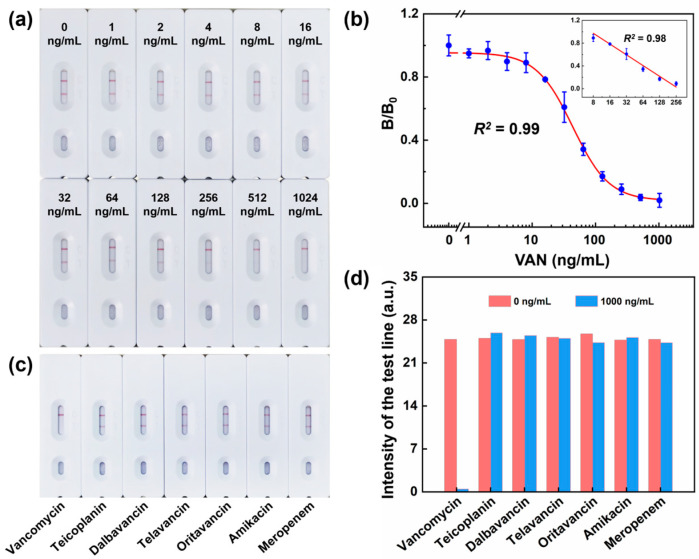
(**a**) Detection limit of colloidal gold test strips assessed by the naked eye. (**b**) A typical calibration curve illustrated by plotting (*B/B*_0_) against VAN concentration; the data are displayed as mean ± standard deviation, *n* = 3. The fitting equation is y = 0.01697 + 0.93724/(1 + (x/43.46265)^1.51219^), with a correlation coefficient (*R*^2^) of 0.99. The top-right inset illustrates the linear portion of calibration curve. The fitting equation is y = 1.5401 − 0.62778 × log_10_(x), with a correlation coefficient (*R*^2^) of 0.98. (**c**,**d**) Cross-reactivity of the test strip with other antibiotics, including teicoplanin, dalbavancin, telavancin, oritavancin, amikacin, and meropenem.

Under optimized assay conditions, we used in-house-prepared VAN standard samples to validate the performance metrics of the test strips. The concentrations of the standard samples were 0, 1, 2, 4, 8, 16, 32, 64, 128, 256, 512, and 1024 ng/mL. For the analysis, we applied 4–5 drops of each sample onto the test strips and allowed them to air-dry for 10 min. We observed an inverse correlation between the VAN concentration and the test line signal intensity. As the concentration of VAN increased, the intensity of the test line signal decreased proportionally, as shown in Figure 3a. We then proceeded to construct a calibration curve to elucidate the correlation between the optical signals (designated as *B/B*_0_) and the respective VAN concentrations. In this context, *B* represents the optical signals for each standard concentration of VAN, while *B*_0_ signifies the optical signal at a zero-concentration baseline. From the calibration curve shown in Figure 3b, illustrated by plotting (*B/B*_0_) against VAN concentration, the fitting equation obtained using Origin software (OriginLab, Northampton, MA, USA) is y = 0.01697 + 0.93724/(1 + (x/43.46265)^1.51219^), with a correlation coefficient (*R*^2^) of 0.99. The top-right inset illustrates the linear portion of calibration curve, where the linear range to detect VAN is 8–256 ng/mL. The fitting equation for this range is y = 1.5401 − 0.62778 × log_10_(x), with a correlation coefficient (*R*^2^) of 0.98.

From the calibration curve, we determined that the LOD, defined as the concentration of VAN giving a 10% inhibition of the maximum absorbance, was 10.3 ng/mL, with an inhibitory concentration 50% (IC_50_) value of 44.5 ng/mL. These values are considerably lower than the concentrations of VAN typically found in the CSF and plasma post administration. This indicates that our VAN test strips are highly applicable for bedside testing of VAN levels in biological systems after drug administration. These strips not only meet clinical requirements but also offer the advantages of simplicity and rapid results, making them an ideal tool for clinical settings. 

### 3.4. Recovery of VAN in the CSF and Plasma of Beagle Dogs

To determine the accuracy of the colloidal gold test strips, the CSF and plasma of beagle dogs containing different concentrations of VAN were analyzed using the test strips. Data representing the mean values of five determinations are summarized in Table 1. Notably, the recovery of VAN from the spiked samples ranges from 87.9% to 106.3%, with the Coefficient of Variation (CV) values ranging from 4.2% to 9.6%, suggesting that the colloidal gold test strips effectively detect the target analyte in CSF and plasma samples.

### 3.5. Specificity of the Colloidal Gold Test Strips

Investigating the cross-reactivity (CR) of colloidal gold test strips is of the utmost importance for evaluating the reliability and specificity of this diagnostic tool. In the present study, we selected four alternative glycopeptide antibiotics—teicoplanin, dalbavancin, telavancin, and oritavancin—as well as two other antibiotics, i.e., amikacin and meropenem, which could show CR. To assess CR, we prepared all the compounds at concentrations of 0, 0.1, 1, 10, 100, 1000, and 10,000 ng/mL and applied them to the test strips. The results, as indicated in Figure 3c,d, show that if a sample contained any antibiotic other than VAN, the test and control lines on the colloidal gold strip turned red. Only samples containing VAN reacted specifically with the colloidal gold–VAN conjugate. The CR values were calculated using the following equation:

CR (%) = [IC_50_ of VAN/IC_50_ of the tested compound] × 100%

wherein the IC_50_ value obtained from the calibration curve represents the concentration at which 50% binding occurs between the antibody and test substances.

The results presented in Table 2 indicate that no CR occurred in the analysis using compounds other than VAN, attributed to the specificity of the antibody for the chemical structure of VAN.

These findings demonstrate that the test strips developed in the present study exhibit high specificity toward VAN. The results show virtually no CR with other potentially interfering compounds, which is essential for a reliable and accurate detection method. This level of specificity ensures that the test can be trusted in clinical settings where quick and accurate results are vital.

### 3.6. Pharmacokinetic Study of VAN in the CSF and Plasma of Beagle Dogs

**Figure 4:**
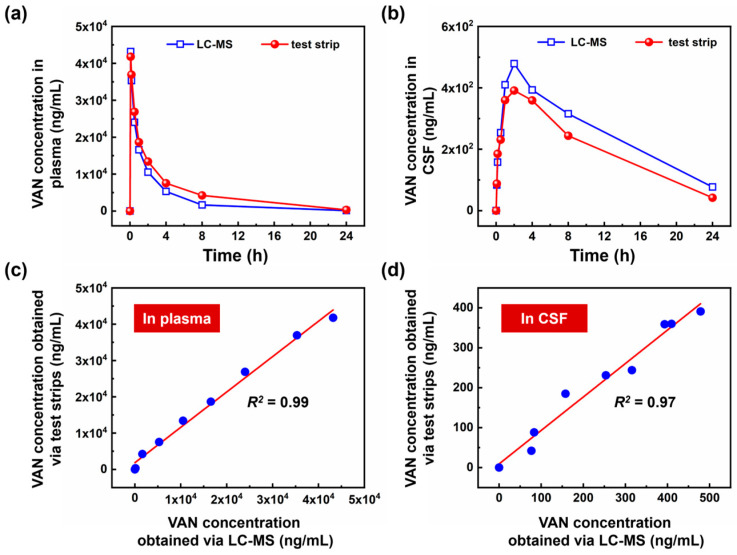
Pharmacokinetic study of vancomycin in beagle dogs over a 24 h period following a 12 mg/kg intravenous injection: (**a**) in plasma and (**b**) in CSF. The blue line represents the results obtained via LC–MS, while the red line indicates the results obtained via the test strip assay. Each data point represents the average VAN concentration of three beagle dogs. The relationship between VAN concentrations obtained from LC-MS and test strips is as follows: (**c**) in plasma, the equation is y = 0.9744x + 1841, with a correlation coefficient (*R*^2^) of 0.99, (**d**) in CSF, the equation is y = 0.8381x + 9.1603, with a correlation coefficient (*R*^2^) of 0.97.

The initial quantification of VAN concentrations in the CSF and plasma samples was conducted using the LC–MS method, yielding a pharmacokinetic curve, as shown in Figure 4a,b (the blue line). This curve delineates the temporal variations in VAN concentrations in the plasma and CSF following intravenous administration in beagle dogs. The data indicate that the concentration of VAN in the plasma peaks rapidly at 40 µg/mL before precipitously dropping to below 1 µg/mL within ~4 h. Meanwhile, the concentration of VAN in CSF ascends gradually, reaching a maximum at ~2 h before declining at a slower rate. Owing to the presence of the blood–brain barrier, the VAN concentration in CSF is much lower than that in plasma.

Further, we subjected the same samples to testing using colloidal gold test strips. Figure 3a reveals that the test line of the test strip vanishes when VAN concentrations exceed 128 ng/mL. For the accurate measurement of such high concentrations, sample dilution is imperative. Based on the LC–MS findings, we adjusted the sample concentrations to a 10–100-ng/mL range, aligning with the linear response range of the colloidal gold test strip. As denoted by the red line in Figure 4a,b, the final results obtained using the test strip agree with those acquired through LC–MS. We investigated the correlation between results obtained from LC-MS and test strips. The results obtained from LC-MS were designated as independent variables, while concentrations determined via the test strip assay served as the dependent variables. To provide a clear visual representation, scatter plots were constructed, depicting the relationship between concentrations obtained from both methods, as illustrated in Figure 4c,d.

Figure 4c offered an in-depth portrayal of vancomycin concentrations in plasma, while Figure 4d showcased those in CSF. The scatter plots revealed a discernible linear relationship, emphasizing the consistency between LC-MS and test strips. For an exhaustive statistical evaluation, regression analyses were conducted using Microsoft Excel. For the plasma samples, the linear regression equation was established as y = 0.9744x + 1841, with a corresponding correlation coefficient (*R*^2^) of 0.99. In terms of the CSF samples, the regression equation was identified as y = 0.8381x + 9.1603, with a correlation coefficient (*R*^2^) of 0.97. Hence, the strong correlation between LC-MS and test strips, apparent in both plasma and CSF samples, underscores the test strip’s potential as a reliable, cost-effective, and time-efficient alternative in clinical diagnostics.

## 4. Conclusions

Herein, we utilized colloidal gold technology to develop a test strip for the rapid detection of VAN in complex biological samples. The test strip achieves high sensitivity for VAN detection through a competitive immunoassay method, with an LOD of 10.3 ng/mL and an IC_50_ value of 44.5 ng/mL. The recovery of VAN from the spiked samples ranged from 87.9% to 106.3%, with the CV values ranging between 4.2% and 9.6%. We evaluated the specificity of the test strip against six types of antibiotics that could potentially show CR, including teicoplanin, dalbavancin, telavancin, oritavancin, amikacin, and meropenem. We found that the colloidal gold test strip demonstrated excellent specificity for VAN, exhibiting no CR (CR < 0.01%) with any antibiotics other than VAN. Finally, we conducted pharmacokinetic tests on three beagle dogs using the test strip, obtaining the concentration curves for VAN in CSF and plasma after administering 12 mg/kg of the medication to the dogs. The results from the test strips closely aligned with those from LC–MS analyses, affirming the test strip’s reliability as an accurate, cost-effective, and swift diagnostic tool suitable for clinical use.

## Figures and Tables

**Table 1:** Recoveries of VAN from spiked CSF and plasma samples.

Sample	Spiked VAN (ng/mL)	Tested VAN (ng/mL)	Recovery (%)	CV (%)
CSF	25	22.0	87.9 ± 8.1	9.2
	50	45.6	91.2 ± 5.9	6.5
	100	106.3	106.3 ± 7.4	7.0
Plasma	25	23.2	92.7 ± 8.9	9.6
	50	49.8	99.5 ± 4.2	4.2
	100	103.7	103.7 ± 5.3	5.1

**Table 2:** Cross-reactivity of the colloidal gold test strip with VAN and other compounds.

Compounds	IC_50_ (ng/mL)	CR (%)
VAN	50	100
Teicoplanin	>10^4^	<0.5
Dalbavancin	>10^4^	<0.5
Telavancin	>10^4^	<0.5
Oritavancin	>10^4^	<0.5
Amikacin	>10^4^	<0.5
Meropenem	>10^4^	<0.5

## Data Availability

Data are contained within the article.
